# Soil metatranscriptome demonstrates a shift in C, N, and S metabolisms of a grassland ecosystem in response to elevated atmospheric CO_2_

**DOI:** 10.3389/fmicb.2022.937021

**Published:** 2022-08-23

**Authors:** David Rosado-Porto, Stefan Ratering, Gerald Moser, Marianna Deppe, Christoph Müller, Sylvia Schnell

**Affiliations:** ^1^Institute of Applied Microbiology, Justus Liebig University, Giessen, Germany; ^2^Faculty of Basic and Biomedical Sciences, Simón Bolívar University, Barranquilla, Colombia; ^3^Institute of Plant Ecology, Justus Liebig University, Giessen, Germany; ^4^School of Biology and Environmental Science and Earth Institute, University College Dublin, Dublin, Ireland

**Keywords:** elevated CO_2_, FACE, functional metatranscriptomics, carbon cycle, nitrogen cycle, sulfur cycle, soil microbiome

## Abstract

Soil organisms play an important role in the equilibrium and cycling of nutrients. Because elevated CO_2_ (eCO_2_) affects plant metabolism, including rhizodeposition, it directly impacts the soil microbiome and microbial processes. Therefore, eCO_2_ directly influences the cycling of different elements in terrestrial ecosystems. Hence, possible changes in the cycles of carbon (C), nitrogen (N), and sulfur (S) were analyzed, alongside the assessment of changes in the composition and structure of the soil microbiome through a functional metatranscriptomics approach (cDNA from mRNA) from soil samples taken at the Giessen free-air CO_2_ enrichment (Gi-FACE) experiment. Results showed changes in the expression of C cycle genes under eCO_2_ with an increase in the transcript abundance for carbohydrate and amino acid uptake, and degradation, alongside an increase in the transcript abundance for cellulose, chitin, and lignin degradation and prokaryotic carbon fixation. In addition, N cycle changes included a decrease in the transcript abundance of N_2_O reductase, involved in the last step of the denitrification process, which explains the increase of N_2_O emissions in the Gi-FACE. Also, a shift in nitrate (${\text{NO}}_{3}^{-}$) metabolism occurred, with an increase in transcript abundance for the dissimilatory ${\text{NO}}_{3}^{-}$ reduction to ammonium (${\text{NH}}_{4}^{+}$) (DNRA) pathway. S metabolism showed increased transcripts for sulfate (${\text{SO}}_{4}^{2-}$) assimilation under eCO_2_ conditions. Furthermore, soil bacteriome, mycobiome, and virome significantly differed between ambient and elevated CO_2_ conditions. The results exhibited the effects of eCO_2_ on the transcript abundance of C, N, and S cycles, and the soil microbiome. This finding showed a direct connection between eCO_2_ and the increased greenhouse gas emission, as well as the importance of soil nutrient availability to maintain the balance of soil ecosystems.

## Introduction

World atmospheric carbon dioxide (CO_2_) concentration has increased by about 50%, from pre-industrial levels of about 278 parts per million volume (ppmV) to the current concentration of more than 415 ppmV (IPCC, 2021; NASA, 2022), and the current anthropogenic emissions of the greenhouse gases (GHG) are the highest in history (IPCC, 2021). Because terrestrial ecosystems act as a “sink” for a significant portion of the global carbon (C), fluctuations in net C exchange between soil and atmosphere impact the CO_2_ concentration in the atmosphere profoundly (DOE.2020, 2020). Hence, the response of terrestrial ecosystems to increasingly higher concentrations of CO_2_ under a changing climate has important implications for the global carbon cycle (Vestergard et al., 2016). In this sense, it has been widely described that elevated CO_2_ (eCO_2_) concentration affects plants in such a way that it decreases transpiration (Owensby et al., 1997; Kimball, 2016) and increases growth (Idso, 1994; He et al., 1995), plant yield (Kimball, 1983), photosynthetic capacity (Habash et al., 1995; He et al., 1995; Johnson and Pregitzer, 2007), below-ground biomass (Jongen et al., 1995), and the efflux amounts of root exudates (Phillips et al., 2012; Jia et al., 2014; Dong et al., 2021).

Consequently, the supply of fresh plant-derived C to the soil matrix due to eCO_2_ may accelerate the decomposition of soil organic matter (SOM) and decrease soil C stocks (Fontaine et al., 2004; Blagodatskaya and Kuzyakov, 2008), a process known as “the priming effect”. This alteration in the increased decomposition of SOM has been previously reported in different ecosystems, such as grasslands (Vestergard et al., 2016; Liu et al., 2017), forests (Phillips et al., 2012; Qiao et al., 2014; Liu et al., 2017), and crop fields (Trivedi et al., 2016). Old SOM pools contain significant physically and chemically protected N stocks; consequently, soil microorganisms under plenty of C supply gain access to a reservoir of N to meet their enhanced N demand (Derrien et al., 2014; Vestergard et al., 2016; Liu et al., 2017), causing alterations in soil N balance and N cycle. This process has been described for the Gi-FACE grassland by Müller et al. (2009), who reported that under eCO_2_, the mineralization of labile organic N became more important. Müller et al. (2009) found that eCO_2_ caused an increase in dissimilatory ${\text{NO}}_{3}^{-}$ reduction to ${\text{NH}}_{4}^{+}$ (DNRA) and immobilization of ${\text{NO}}_{3}^{-}$ and ${\text{NH}}_{4}^{+}$ ions. Other alterations in the N cycle due to eCO_2_ have been described by Kammann et al. (2008), who indicated an increase in N_2_O emissions. Likewise, Moser et al. (2018) reported that N_2_O emissions were 1.79-fold higher for the Gi-FACE grassland under the eCO_2_ treatment. Also, Moser et al. (2018) described that N_2_O emissions from denitrification, nitrification, and heterotrophic nitrification showed a 2.09-fold, 1.64-fold, and 1.66-fold increase, respectively. More recently, Du et al. (2022), based on a meta-analysis, indicated that eCO_2_ significantly increased N_2_O emissions, ${\text{NO}}_{3}^{-}$ content, and soil microbial biomass N by 44, 13, and 7% for agricultural soils.

Likewise, C and N cycle changes are directly related to the soil microbiome and soil microbial processes. For example, Xu et al. (2013) described that the abundance of genes involved in labile C degradation and C and N fixation, and denitrification processes significantly increased under eCO_2_. Similarly, He et al. (2014) and Xiong et al. (2015) have reported a shift in soil microbial communities under eCO_2_ in a soybean and maize agroecosystem, respectively. These changes included stimulation of key functional genes involved in carbon fixation and degradation, nitrogen fixation, denitrification, methane metabolism, and phosphorus cycling. Simonin et al. (2015) reported that shoot biomass, root biomass, and soil respiration were increased under eCO_2_ and N supply, and these variables were positively correlated with the abundance of ammonia-oxidizing bacteria. Le Roux et al. (2016) described that the potential nitrite oxidation rate was enhanced in soil by eCO_2_. Furthermore, the increase in soil microbial C and N cycling may be accompanied by microbial sulfur (S) and phosphorus (P) demand (Xiong et al., 2015; Yu et al., 2018a, 2021). Regarding S cycle alterations under eCO_2_, Yu et al. (2018a,b); Yu et al. (2021) have reported an increase in S cycling in semiarid grassland soils exposed to eCO_2_, indicating a significant increase in the abundance of *dsrA, dsrB*, and *sox* genes. Likewise, Padhy et al. (2020) described that several genera, such as *Desulfatibacillum, Desulfotomaculum, Desulfococcus*, and *Desulfitobacterium*, were more abundant under eCO_2_ conditions in a lowland rice field and that several enzymes involved in S assimilation pathways showed higher counts at eCO_2_ concentrations as well.

Nonetheless, all the above-mentioned studies utilized a DNA-based approach to assessing the changes in the gene abundance involved in the C, N, and S cycles and the microbiome composition under eCO_2_ conditions. DNA-based approaches could lead to biases because DNA from dead cells or free DNA represented a significant fraction of microbial DNA in many soils (Carini et al., 2016). In addition, DNA from dead cells can remain in soils for weeks to years and may cloud DNA-based assessments of microbiome analyses (Dlott et al., 2015; Morrissey et al., 2015). Therefore, using RNA instead of DNA for metastudies provides an ideal tool to study the microbial populations that actively participate in various ecological processes (Sharma and Sharma, 2018). In this sense, some studies were done in the Giessen free-air CO_2_ enrichment experiment (Gi-FACE) in Giessen, Germany, which addressed this issue by performing microbiome metatranscriptomics analyses with rRNA and mRNA. Their findings were that eCO_2_ significantly affected the expression of 16S rRNA, transcription machinery, oxidative phosphorylation, translation and transcription factors, membrane transport, and nucleotide metabolism, among others, associated with rhizosphere microbiomes and plant roots. Likewise, the structure and composition of the rhizosphere soil microbiome were the most affected by eCO_2_ (Bei et al., 2019; Rosado-Porto et al., 2021). Furthermore, these reports showed that through the use of RNA instead of DNA, it was possible to assess the effects of eCO_2_ on the soil microbiome in the Gi-FACE, contrary to the previous studies, which reported little or no effect of it (Regan et al., 2011; de Menezes et al., 2016; Brenzinger et al., 2017).

Nevertheless, in the current literature, the use of mRNA metatranscriptomics to assess the effects of eCO_2_ conditions on C, N, and S cycle processes has not been described. mRNA metatranscriptomics allows addressing which genes are transcribed and to what extent, thereby enabling the demonstration of the functions of a potential range of microorganisms (Franzosa et al., 2014). From such functional data, active metabolic pathways can be identified in the microbiome and can be associated with particular environmental conditions, offering a more informative perspective, as it can reveal details about populations that are transcriptionally active (Bashiardes et al., 2016). Therefore, we hypothesized that the soil metatranscriptome was significantly affected by the eCO_2_ treatment, including the abundance of transcripts involved in nutrient cycling, because of the shifted microbial community under eCO_2_ (Bei et al., 2019; Rosado-Porto et al., 2021). The aims of the present study were: i) to assess the effect of long-term eCO_2_ concentrations on active soil bacteriome, mycobiome, protistome, and virome through an mRNA-based approach; ii) to evaluate the influence of eCO_2_ on C, N, and S cycle expressed genes in a grassland ecosystem; and iii) to propose an interaction model of C, N, and S cycle processes under eCO_2_ conditions.

## Materials and methods

### Study site description

The Gi-FACE study is located at 50°32'N and 8°41.3'E near Giessen, Germany, at an elevation of 172 m above sea level. It consists of three pairs of rings with a diameter of 8 m; each pair consists of an ambient and an elevated CO_2_ treatment ring (Jäger et al., 2003). From May 1998 until the present, eCO_2_ rings have been continuously enriched by 20% above ambient CO_2_ concentrations during daylight hours. Ambient and elevated CO_2_ rings are separated by at least 20 m, and each pair is placed at the vertices of an equilateral triangle. The presence of a slight slope within the experimental site (between 0.5 and 3.5°) places the rings on a moisture gradient, such that pair 1 has the lowest mean moisture content (38.8 ± 10.2%) and pair 2 has the highest mean moisture content (46.1 ± 13.2%), whereas pair 3 is intermediate (40.7 ± 11%) (Jäger et al., 2003; de Menezes et al., 2016). The average annual air temperature and precipitation are 9.4 °C and 580 mm, respectively.

The vegetation is an *Arrhenatheretum elatioris* Br.Bl. *Filipendula ulmaria* subcommunity, dominated by *Arrhenatherum elatius, Galium album*, and *Geranium pratense*. At least 12 grass species, 15 non-leguminous herbs, and up to 5 legumes with small biomass contributions (<5%) are present within a single plot (Andresen et al., 2018). The experimental field has not been plowed for more than 100 years. It has received N fertilization in the form of granular mineral calcium-ammonium-nitrate (40 kg N ha^−1^ year^−1^) once a year since 1995 and has been mown two times a year since 1993. The soil at the Gi-FACE site is classified as Fluvic Gleysol; its texture is a sandy clay loam over a clay layer, with pH = 6.2 and average C and N contents of 4.5 and 0.45%, respectively, as measured in 2001 (Jäger et al., 2003).

### Soil sampling, total RNA extraction, and ribodepletion

Soil sampling was performed utilizing sawed-off 50 ml syringes (11 × 3 cm), and four samples were taken to a depth of ~10 cm within each ring in September 2017. Once taken, samples were refrigerated and transported to the lab for immediate processing. Upon arrival in the laboratory (10-min driving), soil cores were gently shaken by hand to remove loosely attached soil (bulk soil), while the soil that remained attached to the roots was considered rhizosphere soil. Rhizosphere soil was detached from the roots with sterile tweezers and directly sieved. If roots were still present, they were sorted out before sieving. Bulk and rhizosphere soils were sieved (<2 mm) and stored at −80 °C for further analyses. Sample processing took less than 1 h before freezing.

Total RNA extraction was performed following a modified protocol of Mettel et al. (2010), as described by Rosado-Porto et al. (2021). After extraction, samples were treated for DNA digestion with RNase-Free DNase Set (QIAGEN GmbH - Germany) according to the manufacturer's instructions. DNase reaction was stopped with 10 μl of 50 mM EDTA. With the DNA-free RNA, a PCR was carried out, using the universal 16S rRNA gene primers 27F (5'-AGAGTTTGATCMTGGATCMTGGCTCAG-3') and 1492R (5'- GGTTACCTTGTTACGACTT-3') (Lane, 1991; Weisburg et al., 1991) and checked on agarose gel electrophoresis to verify the absence of remaining DNA in the samples as described by Rosado-Porto et al. (2021). Afterward, total RNA technical replicates were pooled into a composite sample according to the ring number and rhizosphere or bulk soil. Later, total RNA samples were ribodepleted using the MICROBExpress™ Kit (Life Technologies, 5791, Carlsbad – California, USA), following the manufacturer's instructions. Finally, mRNA was reverse transcribed to produce double-stranded cDNA (LGC Genomics GmbH, Berlin, Germany).

### cDNA sequencing and metatranscriptomics analysis

The cDNA products were sequenced with Illumina MiSeq V3 (2 × 300 bp), 40 M read pairs/12 Gb of raw data (LGC Genomics GmbH, Berlin, Germany). After sequencing, all libraries for each sequencing lane were demultiplexed using the Illumina bcl2fastq 2.17.1.14 software (Illumina, 2019). Later, sequencing adapter remnants were removed, and reads with a final length of <100 bases were discarded. Afterward, the sequencing outputs were analyzed using SqueezeMeta version 1.3.1 (Tamames and Puente-Sánchez, 2019). Next, sequence assembly was performed using Megahit (Li et al., 2015), and the removal of short contigs (<200 bps) was done with Prinseq (Schmieder and Edwards, 2011). Afterward, RNAs, tRNA/tmRNA, and open reading frames (ORFs) were predicted using Barrnap (Seemann, 2014), Aragorn (Laslett and Canback, 2004), and Prodigal (Hyatt et al., 2010), respectively. Subsequently, Diamond was utilized (Buchfink et al., 2015) to perform the alignment and search of similarities against GenBank (Clark et al., 2016), eggNOG (Huerta-Cepas et al., 2016), and Kyoto Encyclopedia of Genes and Genomes (KEGG) (Kanehisa and Goto, 2000) databases, using an e-value of 10 × 10^−3^ and minimum identity values of 40 and 30 for taxonomical and functional identities, respectively. Additionally, HMM homology searches were done by HMMER3 (Eddy, 2009) for the Pfam database (Finn et al., 2016) applying an e-value of 10 × 10^−10^. Moreover, additional ORFs were produced by Diamond BlastX (Buchfink et al., 2015), implementing an e-value of 10 × 10^−3^ and a minimum identity value of 40. Later, taxonomical classification of the mRNA transcripts was performed using the hits for each query gene utilizing the results of the Diamond search against the GenBank nr database and applying the lowest common ancestor (LCA) algorithm, allowing a 0.2% of different taxa from the LCA and the minimum number of hits per taxa of 2. The read mapping against contigs was performed using Bowtie2 (Langmead and Salzberg, 2012), and the binning was done utilizing MaxBin2 (Wu et al., 2016) and Metabat2 (Kang et al., 2019), and later bin statistics were computed using CheckM (Parks et al., 2015).

### Diversity and differential abundance analyses

For the analysis of SqueezeMeta output, data were imported into R studio software 1.1.419 with package SQMtools version 0.6.1. (Puente-Sánchez et al., 2020). For diversity assessment of bacteria, archaea, fungi, viruses, and protists, frequency tables were created and analyzed with package Phyloseq 1.28.0 (McMurdie and Holmes, 2013). Core features for each of the above taxonomical groups were calculated for eCO_2_ and aCO_2_ conditions by transforming the frequency table counts to relative abundance with Microbiome package version 1.8.0 (Lahti and Shetty, 2019). Later, features with a total relative abundance ≥10 × 10^−4^% and present in ≥95% of samples were included as part of the core. Likewise, for KEGG and GenBank Clusters of Orthologous Groups (COG) protein outputs, features with unknown functions or unassigned names were removed from the frequency tables, and core features were calculated as described above.

For beta diversity analysis, core datasets were transformed using the centered log-ratio (clr) method (Aitchison, 1982, 1986) using the R package ALDEx2 1.22.0 (Fernandes et al., 2013, 2014). Afterward, community dissimilarity distance matrices were generated using the Aitchison distance (Aitchison, 1982, 1986) and visualized using principal components analysis (PCA) (Jolliffe and Cadima, 2016). Statistical differences between CO_2_ conditions were assessed by a permutational multivariate analysis of variance using the Adonis method and employing 999 permutations (Anderson, 2001).

Differential abundance analysis of core features was done with R package ALDEx2 1.22.0 (Fernandes et al., 2013, 2014) by performing the clr transformation using as denominator the geometric mean abundance of all features and 128 Monte Carlo instances. Subsequently, features with absolute ALDEx2 effect sizes of >0.8, >0.5, and >0.2 were considered to have a significantly greater, moderate, and slightly higher abundance, respectively, between aCO_2_ and eCO_2_ rings (Sawilowsky, 2009).

### Pathway reconstruction analysis

Pathway prediction for KEGG (Kanehisa and Goto, 2000) and MetaCyc (Caspi et al., 2018) databases was done using MinPath (Ye and Doak, 2009). In addition, pathway reconstruction and assessment of the log_2_ fold change between aCO_2_ and eCO_2_ rings were performed with SQMtools version 0.6.1. (Puente-Sánchez et al., 2020) and its function “exportPathway” and analyzing feature frequencies as relative abundances.

## Results

### Sequencing and assembly

In total, 23,970,892,090 bases were obtained, comprising 90,534,066 raw sequences, from which 72,253,754 sequences were mapped and assembled with Megahit, with the percentage of sequences successfully mapped per sample ranging between 81.14 and 78.12%. A total of 1,396,973,823 bases from the mapped sequences were retained after short contigs were removed and assembled into 3,997,902 contigs with lengths ranging from 9,714 to 200 bases. From the contigs, there were predicted 4,063,836 ORFs, 1,199,550 rRNAs, and 2,406 tRNAs/tmRNAs, which subsequently were annotated, producing 92,698 successfully annotated taxa and 483,556 and 1,163,975 KEGG and COG annotations, respectively. Details regarding the number of total annotated transcripts per sample are provided in Supplementary material S3.

### Beta diversity and microbe differential abundance

**Figure 1:**
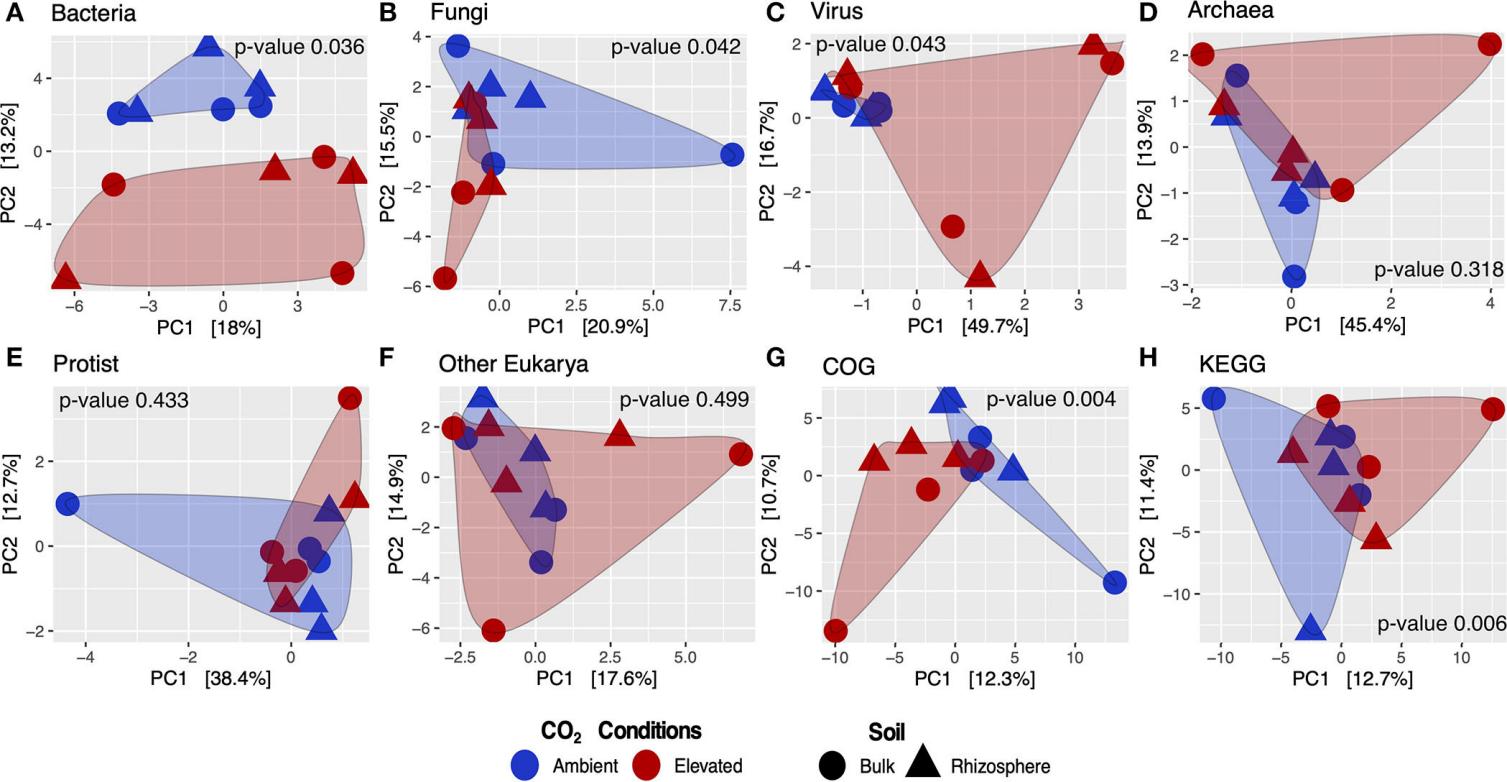
Beta diversity analysis of core microbial taxa and transcripts from the Gi-FACE metatranscriptome. Principal components analysis (PCA) of **(A)** bacteria, **(B)** fungi, **(C)** virus, **(D)** archaea, **(E)** protist, **(F)** other eukarya, **(G)** GenBank COG, and **(H)** KEGG functions; p-values from PERMANOVA test.

Metatranscriptome results from the Gi-FACE exhibited changes in the composition and structure of soil microbial communities due to elevated concentrations of atmospheric CO_2_. Our data indicated that the soil core bacteriome (*p* < 0.05), mycobiome (*p* < 0.05), and virome (*p* < 0.05) were the most affected by eCO_2_ concentrations and showed significantly different compositions between aCO_2_ and eCO_2_ rings, according to the permutational multivariate analysis (Figures 1A–C). In contrast, the general structure of the Gi-FACE soil core archaeome and protistome was not significantly affected by eCO_2_ (Figures 1D,E).

**Figure 2:**
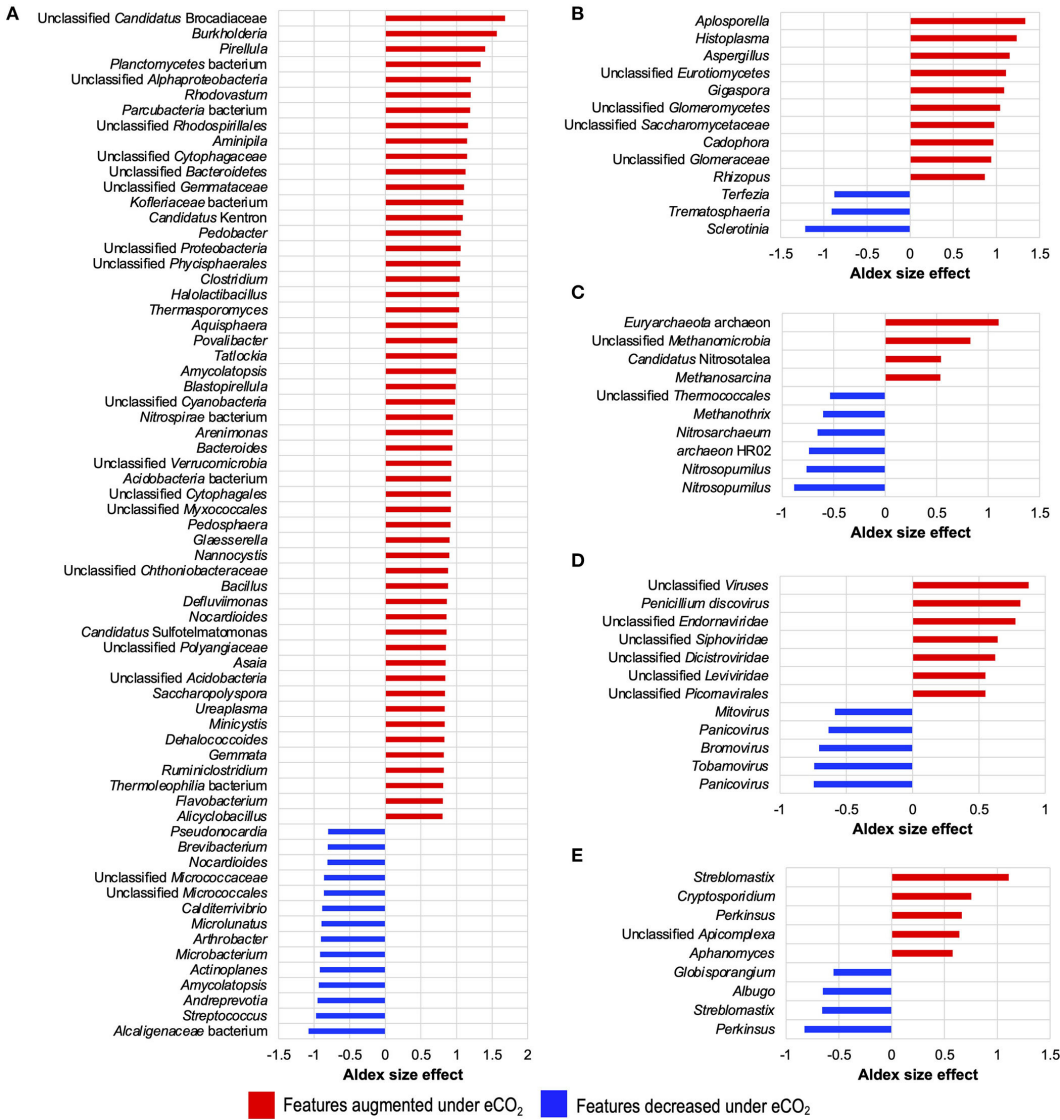
Differential abundance of core microbial taxa from the Gi-FACE metatranscriptome of **(A)** bacteria, **(B)** fungi, **(C)** archaea, **(D)** virus, and **(E)** protist. ALDEx2 results of features with an ALDEx2 effect size > 0.5 using centered log ratio (clr) transformation and the geometric mean abundance of all features.

Moreover, differential abundance results from ALDEx2 (Figure 2) indicated that several taxa were significantly increased or decreased under eCO_2_ conditions and that these affected taxa shaped the soil microbiome of the Gi-FACE. Besides, differential abundance results showed that the number of bacterial taxa that were positively stimulated under eCO_2_ is greater than the number of taxa that were negatively affected. Among the bacterial taxa which were highly stimulated under eCO_2_ conditions are *Flavobacterium, Ruminiclostridium, Gemmata, Dehalococcoides, Minicystis, Ureaplasma, Saccharopolyspora, Asaia, Nocardioides, Defluviimonas, Bacillus, Nannocystis, Glaesserella, Pedosphaera, Arenimonas, Nitrospirae* bacterium, *Blastopirellula, Amycolatopsis, Tatlockia, Povalibacter, Thermasporomyces, Halolactibacillus, Clostridium, Pedobacter, Aminipila, Rhodovastum, Pirellula*, and *Burkholderia*, which showed ALDEx2 effect sizes between 1.5 and 0.8 (Figure 2A, Supplementary material S1.1). Likewise, soil mycobiome was shaped by several fungi greatly affected under eCO_2_ conditions, most belonging to phyla Basidiomycota, Mucoromycota, and Ascomycota, as is the case of the genus *Aspergillus* (phylum *Ascomycota*), which showed an ALDEx2 effect size of 1.15 (Figure 2B, Supplementary material S1.2). Additionally, fungi like *Rhizopus, Cadophora, Gigaspora, Histoplasma*, and *Aplosporella* were also highly stimulated in eCO_2_ rings with effect sizes from 0.86 to 1.33 (Figure 2B). Regarding the Gi-FACE soil virome, viruses like Brome mosaic virus, Panicovirus, and Cocksfoot mild mosaic virus decreased in eCO_2_ rings presenting effect sizes ranging from −0.58 to −0.74. In contrast, viruses such as *Penicillium discovirus*, unclassified *Picornavirales*, and unclassified *Endornaviridae* were positively affected under eCO_2_ conditions with effect sizes from 0.54 to 0.87 (Figure 2D, Supplementary material S1). Moreover, some viral features belonging to the families Leviridae, Siphoviridae, Bromoviridae, and Dicistroviridae were affected by eCO_2_ as well (Supplementary material S1.3).

Although our data did not show that eCO_2_ significantly influenced the general structure of the soil archaeome and protistome, the differential abundance test showed that some archaea and protist taxa were either positively or negatively affected under eCO_2_ conditions (Figures 1D,E, 2C,E, Supplementary materials S1.4, S1.5).

### Functional metatranscriptome and differential abundance

Beta diversity of transcripts analyzed against GenBank, COG, and KEGG databases showed that the functional metatranscriptome was greatly affected under eCO_2_ conditions in which the annotations performed to both databases were significantly different in their structure and composition between eCO_2_ and aCO_2_ conditions (Figures 1G,H). After removing unclassified and non-characterized proteins, 7,780 remained for GenBank COG and 8,880 for KEGG datasets. Furthermore, our data indicated that the sequences analyzed against both databases showed similar results regarding the number of proteins with an ALDEx2 effect size greater than 0.5. In the case of GenBank COG data, 146 transcripts were moderately or greatly stimulated under eCO_2_ conditions, in contrast to 161 negatively affected under these conditions. Similarly, KEGG results showed that the abundance of 147 and 156 transcripts was positively and negatively affected, respectively (Supplementary material S2.1).

**Figure 3:**
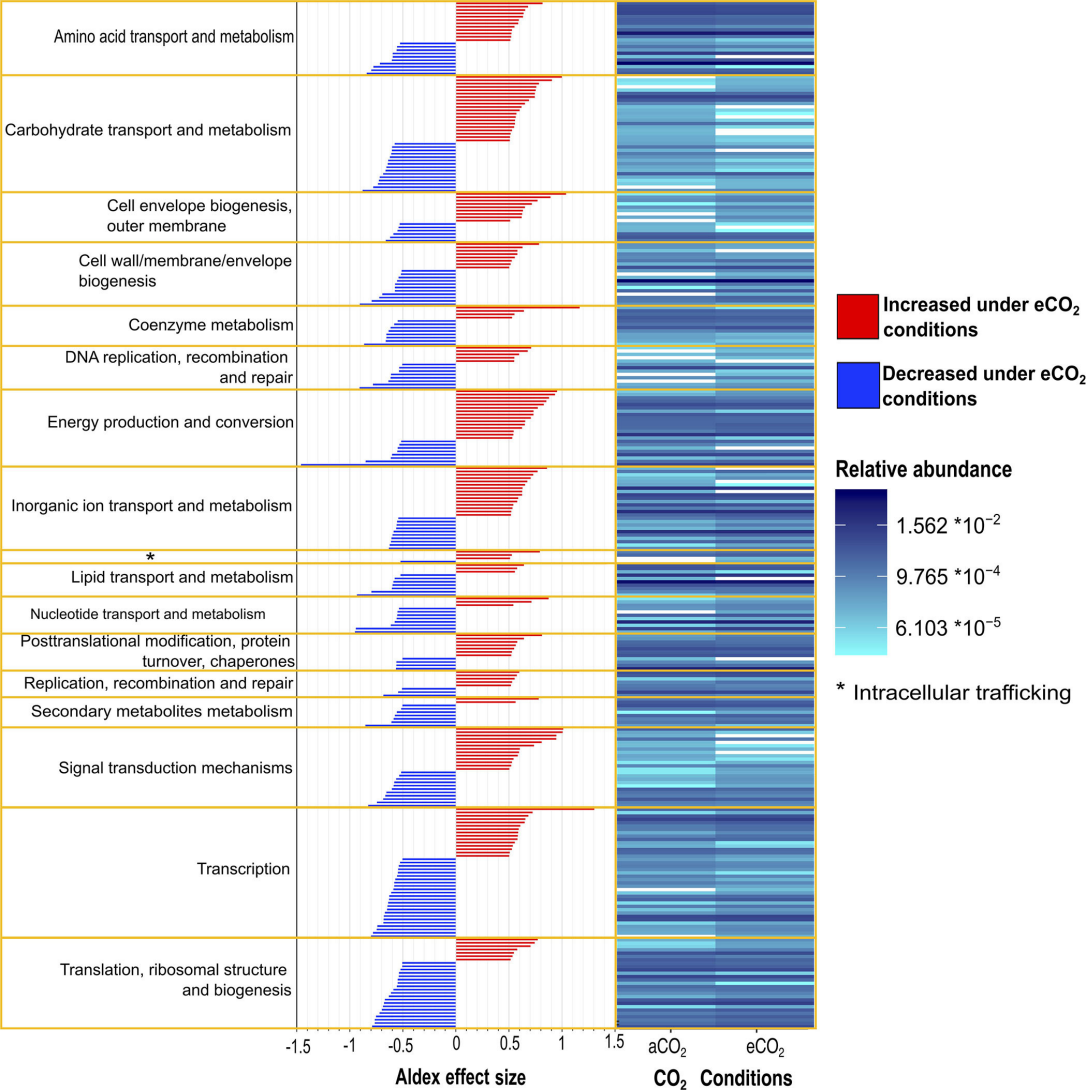
Differential abundance of Gi-FACE metatranscriptome core transcripts annotated against GenBank Clusters of Orthologous Groups (COG) and grouped according to COG categories. Results expressed as relative abundance **(right)** and ALDEx2 effect size **(left)** of transcripts with an ALDEx2 effect size > 0.5 using centered log ratio (clr) transformation and the geometric mean abundance of all features.

Moreover, eCO_2_ conditions positively influenced the transcript abundance of several COG categories, such as energy production and conversion, inorganic ion transport and metabolism, cell envelope biogenesis, outer membrane intracellular trafficking carbohydrate transport and metabolism, and signal transduction mechanisms Figure 3, (Supplementary material S2.2). In contrast, categories for translation, ribosomal structure, and biogenesis; transcription; secondary metabolite biosynthesis, transport, and catabolism; nucleotide transport and metabolism; DNA replication, recombination, and repair; and coenzyme metabolism were negatively affected at eCO_2_ concentrations (Figure 3, Supplementary material S2.2).

### Nitrogen cycle

**Figure 4:**
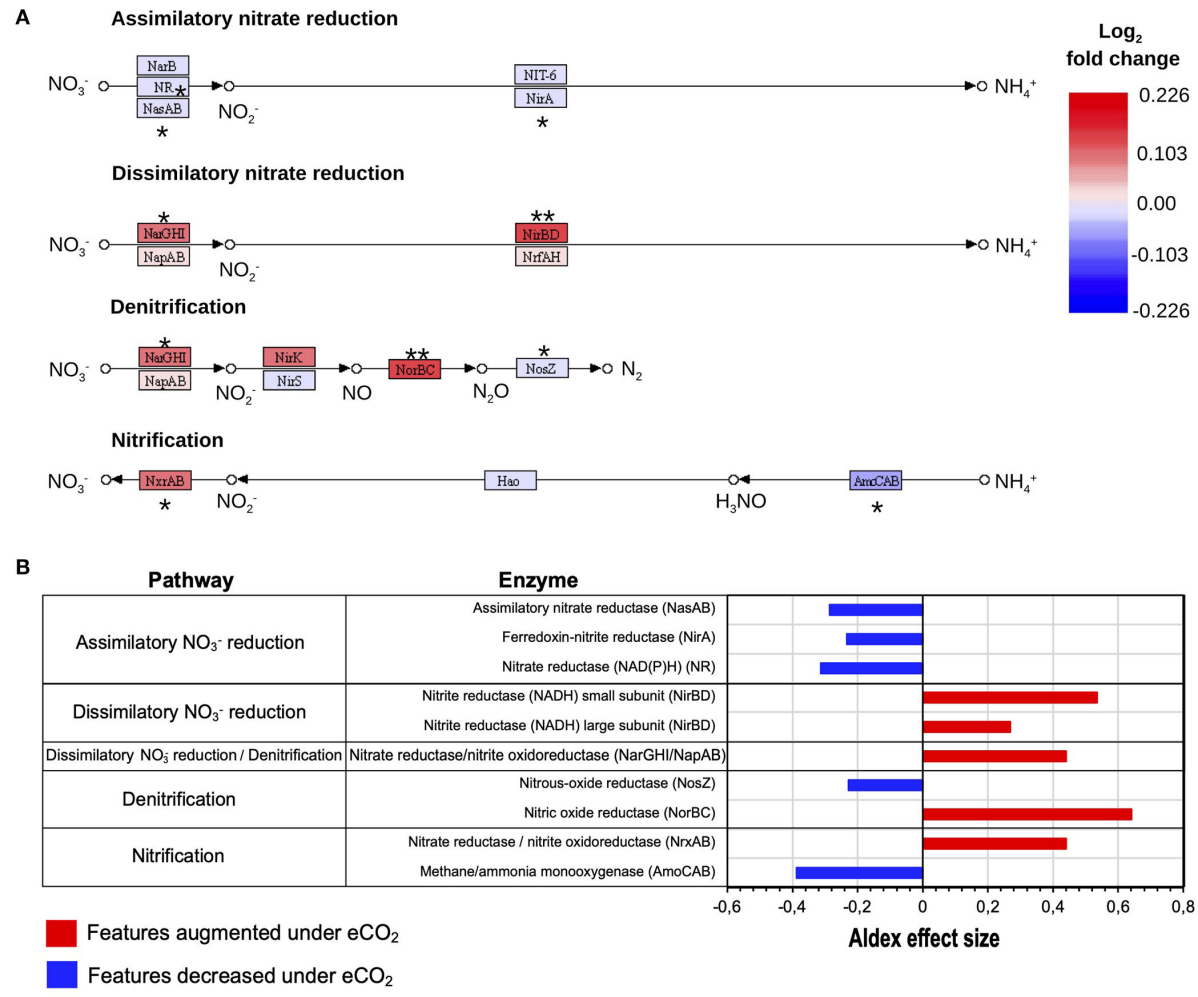
Reconstructed KEGG (Kanehisa and Goto, 2000) N pathways of ${\text{NO}}_{3}^{-}$ assimilatory and dissimilatory reduction, denitrification, and nitrification processes. **(A)** N transformations expressed as log_2_ fold change of transcripts as relative abundance. ALDEx2 effect size: (**) >0.5, (*) >0.2. **(B)** Differential abundance of N cycle transcripts with ALDEx2 effect sizes > 0.2.

The data showed that, under eCO_2_ conditions, an increase in the dissimilatory nitrate (${\text{NO}}_{3}^{-}$) reduction to ammonium (${\text{NH}}_{4}^{+}$) (DNRA) pathway and a decrease in the assimilatory ${\text{NO}}_{3}^{-}$ reduction to ${\text{NH}}_{4}^{+}$ occurred (Figure 4A). Specifically, the transcripts for the DNRA enzymes nitrite reductase (NADH) (NirBD) and nitrate reductase (NarGHI) showed greater abundances under eCO_2_ conditions (Figures 4A,B). In contrast, the transcript abundance for the enzymes nitrate reductase (NAD(P)H) (NR), ferredoxin-nitrite reductase (NirA), and assimilatory nitrate reductase (NasAB) was negatively affected at eCO_2_ concentrations (Figures 4A,B, Supplementary material S2.3).

Similarly, the denitrification process showed changes as well. Transcript abundance for the denitrification enzymes nitrate reductase/nitrite oxidoreductase (NarGHI/NapAB) and nitric oxide reductase (NorBC) showed higher levels under eCO_2_ conditions with ALDEx2 effect sizes of 0.44 and 0.64. These enzymes are responsible for the transformation of ${\text{NO}}_{3}^{-}$ to nitrite (${\text{NO}}_{2}^{-}$) and the reduction of nitric oxide (NO) to nitrous oxide (N_2_O), respectively. On the contrary, the transcription of the gene for the enzyme nitrous oxide reductase (NosZ), which catalyzes the transformation of N_2_O to atmospheric nitrogen (N_2_), was reduced in the eCO_2_ rings (Figures 4A,B, Supplementary material S2.3).

Likewise, the nitrification process was also affected. The data exhibited changes in the expression patterns of the enzymes methane/ammonia monooxygenase (AmoCAB) and nitrate reductase/nitrite oxidoreductase (NrxAB), which were negatively and positively affected, respectively (Figures 4A,B, Supplementary material S2.3). Furthermore, pathway reconstruction and differential abundance analyses did not show significant changes in the abundance of N fixation enzymes under eCO_2_ conditions.

### Sulfur cycle

**Figure 5:**
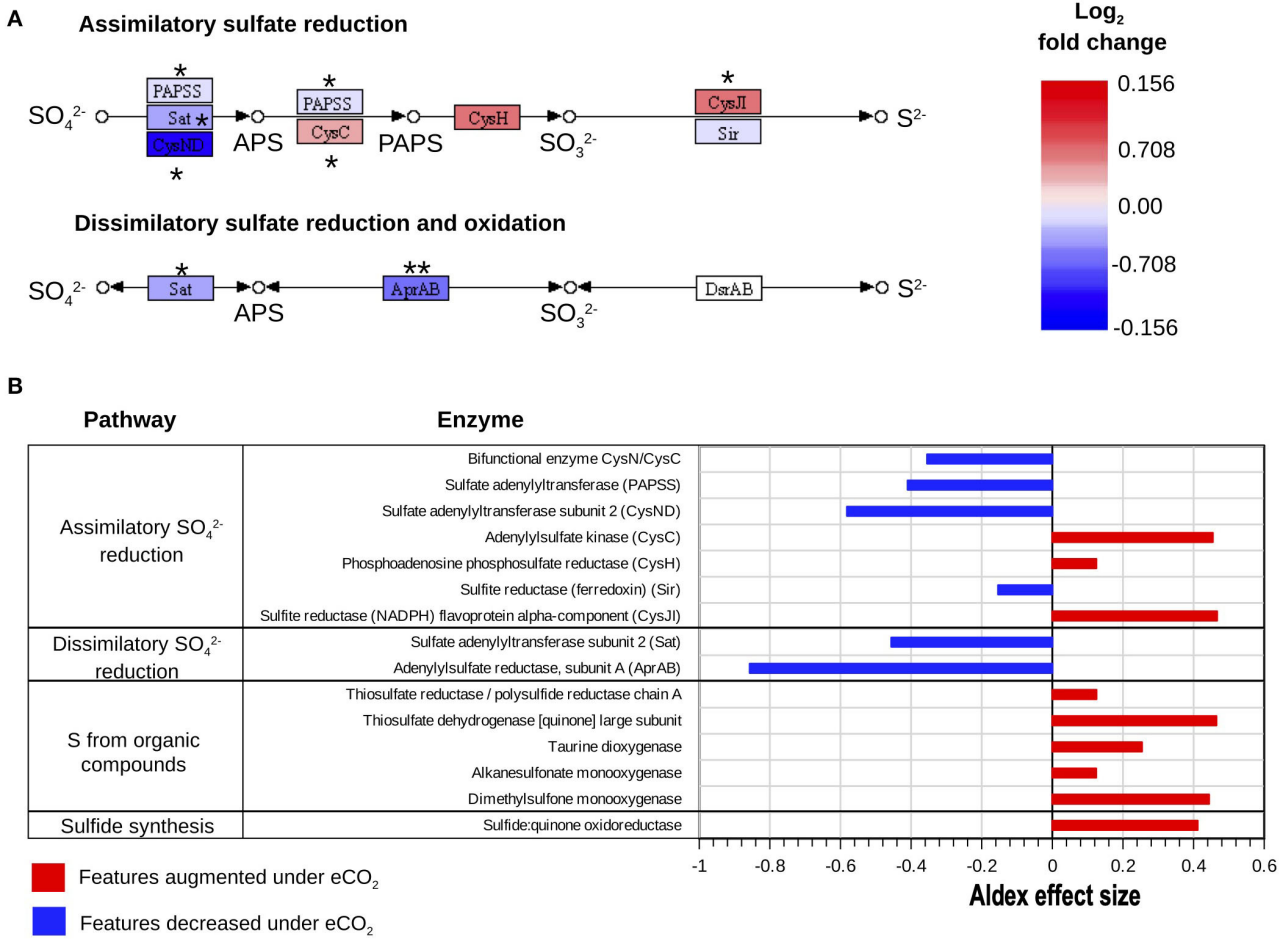
Reconstructed KEGG (Kanehisa and Goto, 2000) pathways of S metabolism. **(A)** Assimilatory ${\text{SO}}_{4}^{2-}$ reduction and dissimilatory ${\text{SO}}_{4}^{2-}$ reduction and oxidation processes expressed as log_2_ fold change of transcripts as relative abundance. ALDEx2 effect size: (***) >0.8, (**) >0.5, (*) >0.2. **(B)** Differential abundance of S cycle transcripts with absolute ALDEx2 effect sizes > 0.1 involved in assimilatory ${\text{SO}}_{4}^{2-}$ reduction, dissimilatory ${\text{SO}}_{4}^{2-}$ reduction, and oxidation, and uptake of S from organic compounds and sulfide synthesis.

The metatranscriptomics results indicated changes in the dissimilatory and assimilatory pathways of sulfate (${\text{SO}}_{4}^{2-}$) reduction. Transcript abundance for the enzymes sulfate adenylyltransferase (Sat) and adenylylsulfate reductase (AprAB), part of the dissimilatory ${\text{SO}}_{4}^{2-}$ reduction pathway, was highly decreased under eCO_2_ conditions. Furthermore, the transcript abundance for the enzyme AprAB was the one that showed the highest decrease, with an ALDEx2 effect size of −0.86. This protein catalyzes the transformation of sulfite (${\text{SO}}_{3}^{2-}$) to adenosine 5'-phosphosulfate (APS) (Figures 5A,B).

Similarly, the decrease in transcript abundance of the sulfate adenylyltransferase subunit 2 (CysND) and sulfate adenylyltransferase (PAPSS), part of the assimilatory sulfate reduction pathway, suggested a decrease in the reduction of ${\text{SO}}_{4}^{2-}$ to APS at eCO_2_ concentrations. These two proteins showed ALDEx2 effect sizes of −0.58 and −0.41, respectively (Figures 5A,B, Supplementary material S2.4). Nonetheless, the enzymes adenylylsulfate kinase (CysC) and sulfite reductase (NADPH) (CysJI), which are involved in the reduction of APS to 3′-Phosphoadenosine-5′-Phosphosulfate (PAPS) and the reduction of ${\text{SO}}_{3}^{2-}$ to sulfide (S^2−^), respectively, were increased at eCO_2_ concentrations (Figures 5A,B, Supplementary material S2.4).

Moreover, our data showed that several enzymes belonging to pathways responsible for the transformation of organic S compounds had higher transcript abundances at eCO_2_, as is the case with dimethylsulfone monooxygenase, thiosulfate dehydrogenase [quinone], and taurine dioxygenase (Figure 5B, Supplementary material S2.4). Although the SOX system for the oxidation of S was generally not over-expressed under eCO_2_ concentrations, transcripts of the enzyme sulfane dehydrogenase subunit (SoxC) showed a slight increase at these conditions, with an ALDEx2 effect size of 0.28.

### Carbon cycle

**Figure 6:**
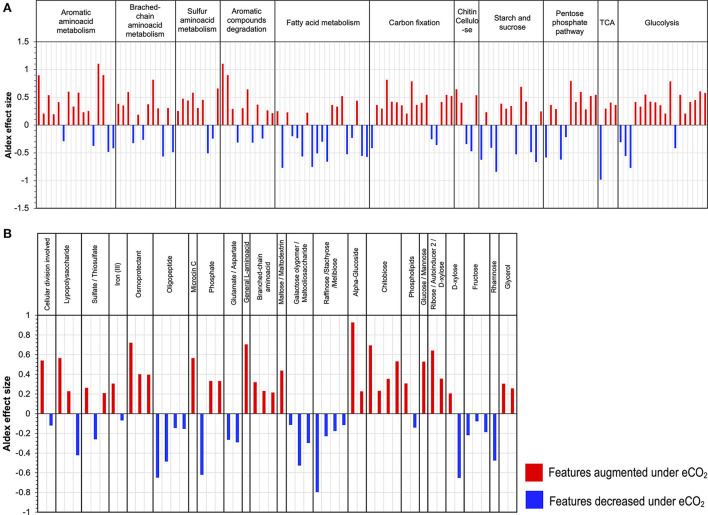
Differential abundance of transcripts grouped by KEGG Orthology (KO) second level of **(A)** carbon compounds metabolism and **(B)** ABC transporters.

Functional metatranscriptome showed changes in the metabolism of C compounds. The main changes comprised a general increase in transcripts from the glycolytic and pentose phosphate pathways, which included the increase in the abundance of transcripts for the enzymes phosphoglucomutase, glucose-6-phosphate isomerase, phosphoenolpyruvate carboxykinase (ATP), pyruvate water dikinase, 2-oxoglutarate, gluconate 2-dehydrogenase, gluconolactonase, transketolase, and xylulose-5-phosphate/fructose-6-phosphate phosphoketolase, all with ALDEx2 effect sizes ranging from 0.79 to 0.52 (Figure 6A). Likewise, the data exhibited an increase in the transcription of genes coding for enzymes responsible for the degradation of chitin, cellulose, and aromatic compounds, for example, alpha-N-arabinofuranosidase; endo-1,4-beta-xylanase, and chitinase (Figure 6A). In contrast, the metabolism of fatty acids, starch, and sucrose was negatively affected under eCO_2_ conditions, with the most affected features having ALDEx2 effect sizes ranging from −0.84 to −0.51 (Figure 6A, Supplementary material S2.5).

Furthermore, our results indicated a stimulation in the metabolism of aromatic, branched-chain, and sulfur amino acids. In the case of sulfur amino acid metabolism, an increase in the transcript abundance of enzymes for the metabolism of homocysteine, taurine, and thiol groups occurred (Supplementary material S2.5). Likewise, the transcript abundance of several enzymes involved in the degradation of aromatic amino acids was highly stimulated, for example, aminocarboxymuconate-semialdehyde decarboxylase case enoyl-CoA hydratase, amidase, monoamine oxidase, acylpyruvate hydrolase, and gentisate 1,2-dioxygenase, with ALDEx2 effect sizes between 1.10 and 0.54. Moreover, the transcript abundance for genes involved in the Arnon–Buchanan cycle (reductive citric acid cycle) increased under eCO_2_, including key enzymes like phosphoenolpyruvate carboxykinase (ATP), pyruvate water dikinase, and pyruvate ferredoxin oxidoreductase (Figure 6A, Supplementary material S2.5).

### ABC membrane transporters

The metatranscriptomic data on the ABC membrane transport proteins suggested changes in the uptake and transport of different carbon compounds under eCO_2_ conditions. For example, membrane transporters for glucose/mannose, α-glucoside, ribose/D-xylose, and chitobiose increased at eCO_2_ concentrations. In contrast, there was a decrease in the expression of membrane transporters for raffinose/stachyose/melibiose, rhamnose, galactose oligomer/maltooligosaccharide, maltose, and fructose (Figure 6B, Supplementary material S2.6). Similarly, a shift in the ABC transporters for amino acids occurred, with an increase in the transcript abundance of the transporters for general L-amino acids and branched-chain amino acids and a decrease in glutamate/aspartate and oligopeptide transporters (Figure 6B, Supplementary material S2.6). Additionally, other membrane transport proteins over-expressed under eCO_2_ conditions, including transporters for osmoprotectants, lipopolysaccharides, and iron (II) (Figure 6B, Supplementary material S2.6).

## Discussion

### Soil microbiome response to eCO_2_

Our results on the functional metatranscriptome of the Gi-FACE confirm previous reports from Bei et al. (2019) and Rosado-Porto et al. (2021) on the changes in microbiome composition due to eCO_2_ concentrations. Additionally, the outcome expands our understanding of eCO_2_ concentrations in N, S, and C cycles. The metatranscriptome data presented in this study are a one-time snapshot of the gene expression of the microbiome. Although transcriptomes are dynamic and variable in response to diverse environmental factors (Nuccio et al., 2021; Zhao et al., 2021), it nevertheless provides valuable information on soil ecosystems' response to climate change scenarios.

Our data confirm that the structure of the Gi-FACE soil bacteriome was strongly affected under eCO_2_ (Figure 1A). Prior studies have already portrayed differences in the composition of the bacteriomes between aCO_2_ and eCO_2_ conditions (Bei et al., 2019; Rosado-Porto et al., 2021), who have described significant changes mainly in the rhizosphere. Additionally, several bacterial taxa found in the present study (Figure 2A, Supplementary material S1.1) have already been described as stimulated under eCO_2_ conditions, as is the case of genera *Bacillus, Burkholderia, Mesorhizobium, Streptomyces*, and *Dongia* (Rosado-Porto et al., 2021). Besides the soil bacteriome, the results showed that the soil mycobiome was greatly affected at eCO_2_ concentrations. Like Bei et al. (2019), our data indicated that the Gi-FACE mycobiome was composed mainly of phyla Basidiomycota, Mucoromycota, and Ascomycota (Supplementary material S1.2). Moreover, several highly affected fungi belonged to Ascomycota families, such as Mycosphaerellaceae, Didymosphaeriaceae, Ophiocordycipitaceae, Saccharomycetaceae, and Aspergillaceae, and Mucoromycota families, such as Cunninghamellaceae, Rhizopodaceae, and Glomeraceae (Supplementary material S1.2). Nevertheless, although our results showed a significant effect of eCO_2_ concentrations on the mycobiome composition, the reports of its effect on soil fungal communities vary according to different authors. Carney et al. (2007) described a decrease in fungal abundance under eCO_2_ conditions, whereas some others reported no significant change in the fungal communities (He et al., 2010; Hayden et al., 2012). This indicates that the response of fungal communities to eCO_2_ depends on other environmental factors like temperature and soil moisture and may be ecosystem specific as well.

The effects of CO_2_ concentration on the metagenome or metatranscriptome of soil archaeomes have not been widely studied; however, some reports described a strong influence of CO_2_ concentrations on soil archaeal communities (Hayden et al., 2012; Lee et al., 2015; Lee and Kang, 2016). Although the Gi-FACE archaeome did not show significant differences in its structure and composition in response to eCO_2_ concentrations, some taxa showed changes in their abundance (Figure 2C, Supplementary material S1), most belonging to the family Nitrosopumilaceae (phylum Thaumarchaeota). In addition, in the present study, the core archaeome was mainly composed of the phylum Euryarchaeota, contrary to that reported by Bei et al. (2019), who reported the phylum Thaumarchaeota as the most abundant.

Furthermore, our data exhibited that alongside the soil bacteriome and mycobiome, the soil core virome was affected by the eCO_2_ in the Gi-FACE (Figure 2D, Supplementary material S1.3). So far, in the current literature, there are no reports about the effects of eCO_2_ on the soil virome. Moreover, some reports indicated that, in general, the diversity of the soil virome is highly underestimated, with most of the current information focused on bacterial phages, while almost nothing is known about viruses that infect non-bacterial soil microbes, such as the archaea, fungi, and soil protozoa (Williamson et al., 2017; Pratama and van Elsas, 2018). Our results on the differential abundance of the core virome under eCO_2_ conditions suggested that several viral transcripts were reacting to changes in bacterial, archaeal, and fungal taxa. For example, the Leviridae and Siphoviridae families include viruses that use bacteria and archaea as hosts and in our study were increased under eCO_2_ conditions (Supplementary material S1.3) (Duin and Olsthoorn, 2012; Hendrix et al., 2012; Krupovic et al., 2020). Similarly, some fungal viruses, such as *Mitovirus* and *Penicillium discovirus*, have shown significant changes in their abundance under eCO_2_ conditions, which might be linked to the changes in some fungal features as observed in *Penicillium oxalicum* and members belonging to the class Ophiostomatales (Hong et al., 1999; Krishnamurthy, 2017).

### Changes in C compound assimilation and priming effect

**Figure 7:**
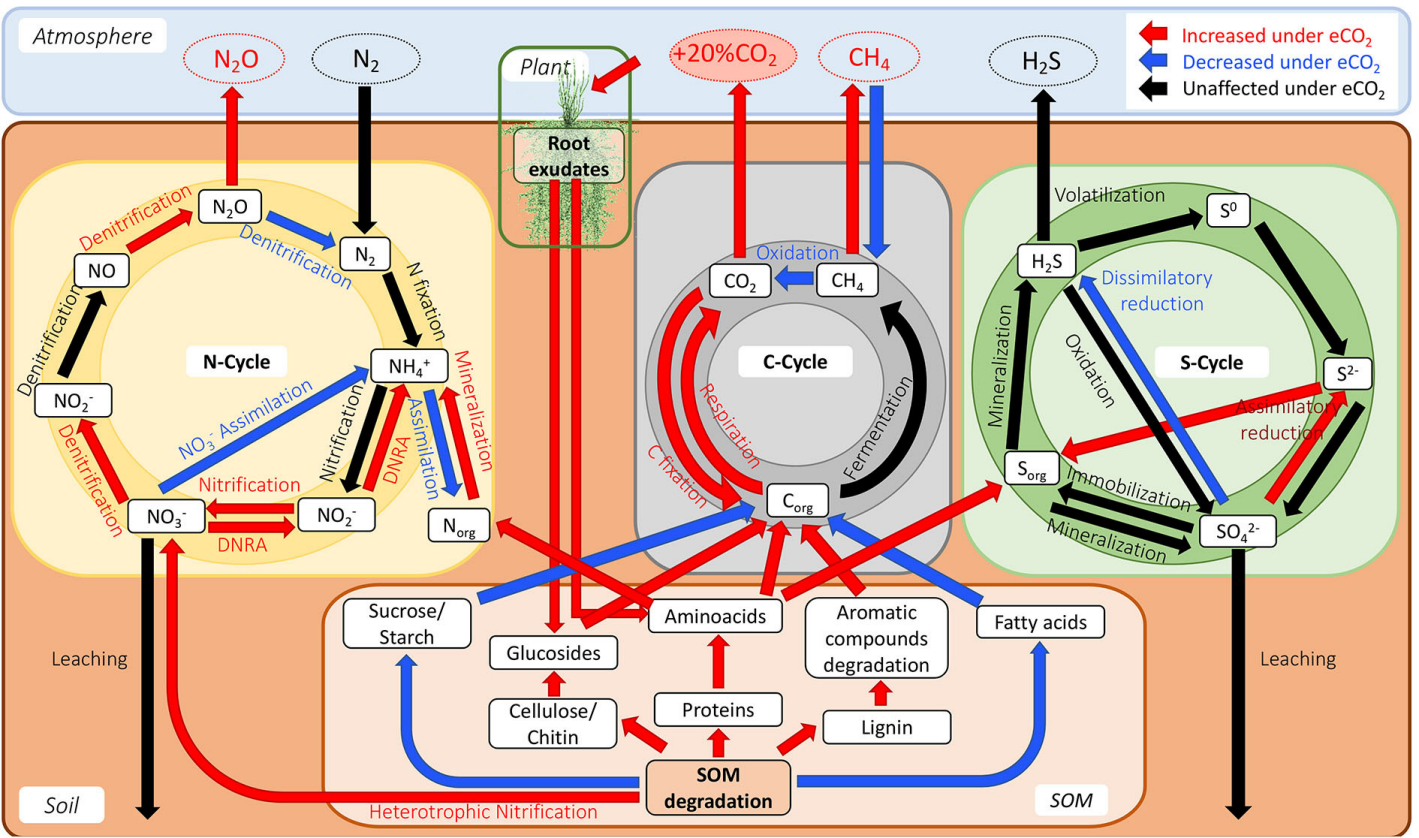
Model diagram of the interaction of C, N, and S cycles in the Gi-FACE.

The data showed changes in transcript abundance from pathways involved in the metabolism of different C compounds, indicating that C dynamics have changed due to eCO_2_. It has been widely described that eCO_2_ increases the efflux of soluble sugars, amino acids, phenolic acids, and organic acids in the root exudates (Phillips et al., 2012; Jia et al., 2014; Dong et al., 2021), which produces the so-called “priming effect,” thus leading to an acceleration in SOM decomposition (Fontaine et al., 2004; Blagodatskaya and Kuzyakov, 2008). The metatranscriptome data showed an increase in the priming effect due to eCO_2_ concentrations in the Gi-FACE soil. The priming effect in our data is represented mainly by the over-expression of transcripts from the glycolysis and pentose phosphate pathways and an increase in transcript abundance for certain amino acid metabolism, alongside an increase in transcript abundance of enzymes responsible for the degradation of chitin, cellulose, and lignin (Figure 7). Similarly, He et al. (2010, 2014), Xiong et al. (2015), and Yu et al. (2018a,b) have reported the stimulation of functional gene abundance for C compound degradation, either labile or recalcitrant under eCO_2_. Likewise, other authors have described the increase in the degradation of soil organic polymers as part of the decomposition of older soil C (Van Groenigen et al., 2005; Xie et al., 2005; Niklaus and Falloon, 2006; Vestergard et al., 2016). This enhancement of carbohydrate, amino acid, and SOM degradation would be reflected in a higher respiration rate and, consequently, a higher efflux of CO_2_. In our previous study, CO_2_ soil fluxes were 35% higher in eCO_2_ rings compared to the aCO_2_ ones (Rosado-Porto et al., 2021).

Furthermore, the data suggested a shift in the uptake and use of C sources at eCO_2_ concentrations, reflected in a shift toward higher utilization of sugars and amino acids and a decrease in the metabolism of lipids, especially fatty acids (Figures 3, 6A). Additionally, the analysis of ABC membrane transporters revealed changes in the transcript abundance for saccharide uptake systems that are more often used under eCO_2_ conditions, indicating a shift in preference for the uptake of glucose, mannose, α-glucosides, ribose, xylose, and chitobiose instead of raffinose, stachyose, melibiose, rhamnose, galactose, maltose, and fructose. Moreover, our results revealed an increase in transcripts for prokaryotic carbon fixation at eCO_2_ concentrations. This included increases in enzymes, such as phosphoenolpyruvate carboxykinase, pyruvate water dikinase, and pyruvate ferredoxin oxidoreductase, accompanied by a decrease in the ribulose-bisphosphate carboxylase (Rubisco) (Figure 6A, Supplementary material S2.5). These results are opposite to the ones reported by He et al. (2010, 2014), Xu et al. (2013), Xiong et al. (2015), and Yu et al. (2018a,b), who described a significant increase in the gene abundance of the Rubisco enzyme under eCO_2_ conditions. The aforementioned results suggest that in the Gi-FACE, the C fixation performed by prokaryotes at eCO_2_ concentrations is very likely to be done through the reverse reductive citric acid cycle, also known as the Arnon–Buchanan cycle (Evans et al., 1966; Buchanan and Arnon, 1990; Buchanan et al., 2017) instead of the Rubisco pathway. The increase in transcript abundance of the described enzymes involved in the reverse citric acid cycle could be associated with the increase of some members of the Nitrospirae and Aquificae phyla, which were significantly augmented under eCO_2_ (Figure 2, Supplementary material S1.1) (Berg, 2011; Alfreider et al., 2018; Mundinger et al., 2019; Steffens et al., 2021). Nonetheless, it is difficult to differentiate CO_2_ fixation for autotrophic growth from anapleurotic reactions of the citric acid cycle. Moreover, this autotrophic pathway might be more widespread among anaerobic and microaerobic bacteria (Berg, 2011) and possibly in aerobic bacteria (Buchanan et al., 2017).

### Shift in N cycle processes

Changes in the N cycle have been previously described in the Gi-FACE (Kammann et al., 2008; Müller et al., 2009; Moser et al., 2018). However, the underlying microbiological mechanisms driving these processes were not detected until now. The metatranscriptomic results confirmed a switch in the ${\text{NO}}_{3}^{-}$ reduction at eCO_2_ concentrations, from an assimilatory process to a DNRA, reflected by the increase in transcript abundance of the enzymes nitrite reductase (NADH) (NirBD) and nitrate reductase (NarGHI), responsible for the transformations of ${\text{NO}}_{3}^{-}$ to ${\text{NO}}_{2}^{-}$ and from ${\text{NO}}_{2}^{-}$ to ${\text{NH}}_{4}^{+}$ in the DNRA process (Figures 4A,B, 7). Previously, Müller et al. (2009), utilizing a ^15^N labeling approach, identified an increase in the DNRA and the immobilization of ${\text{NH}}_{4}^{+}$ and ${\text{NO}}_{3}^{-}$.

Additionally, our functional metatranscriptomic approach gives some clarity about the processes leading to the excess production of N_2_O under eCO_2_ conditions previously described by Kammann et al. (2008), Moser et al. (2018), and more recently in the meta-analysis of Du et al. (2022). The data suggest that the alteration in the denitrification process leads to an increase in N_2_O production. This increase results from over-expression of the N_2_O-producing enzyme nitric oxide reductase (NorBC) and a reduced expression of the enzyme nitrous oxide reductase (NosZ), which lead to a decrease in N_2_O reduction to N_2_. Furthermore, results indicate that the increase in N_2_O is caused both by an increase in N_2_O production and a decrease in N_2_O reduction (Figure 7). These results seem to denote that changes in nitrous oxide reductase (NosZ) occur at a transcriptional level. Contrary to our data, previous reports from Liu et al. (2010) and Bakken et al. (2012) indicated that the high N_2_O:N_2_ product ratio is a post-transcriptional phenomenon only due to the sensitivity of this enzyme to lower pH values that are usually found at eCO_2_ concentrations and therefore affect its translation/assembly.

The results also showed changes in the nitrification process, represented by a reduction in transcripts involved in the conversion of ${\text{NH}}_{4}^{+}$ to hydroxylamine (H_3_NO) performed by the enzyme methane/ammonia monooxygenase (AmoCAB), accompanied by an increase in the transcript abundance for the enzyme nitrate reductase/nitrite oxidoreductase (NrxAB), suggesting an increase in the rate of transformation from ${\text{NO}}_{2}^{-}$ to ${\text{NO}}_{3}^{-}$ (Figures 4A,B, 7). Additionally, a reduction in the transcript abundance for the first nitrification step was observed, which denotes that under eCO_2_ conditions, soil organisms obtain N from other sources instead of ${\text{NH}}_{4}^{+}$. Previously, Müller et al. (2009) described that the mineralization of labile organic N became more critical at eCO_2_ concentrations.

The changes in transcript abundance for the nitrification process might suggest an increase in heterotrophic nitrification by fungi. The fungal nitrification comprises the oxidation of different forms of organic N, such as L-asparagine, propionamide, malonylmonohydroxamate, and 3-nitropropionate, using peroxidase enzymes (Hora and Iyengar, 1960; Marshall and Alexander, 1962; Doxtader and Alexander, 1966). More recently, Laughlin et al. (2008) and Zhu et al. (2015) have described that fungi carried out a significant part of the nitrification in soils and that they can simultaneously oxidize ${\text{NH}}_{4}^{+}$ and organic N. Moreover, many of the fungal taxa that are able to perform nitrification are members of the genus *Aspergillus* (Hora and Iyengar, 1960; Marshall and Alexander, 1962; Doxtader and Alexander, 1966), one of the most positively affected in the Gi-FACE mycobiome (Figure 2B). Therefore, the above-mentioned results might support the idea of the mining of SOM by soil microorganisms, very likely fungi, in order to fulfill their N requirements under eCO_2_, and could be linked to the previously reported data of higher C:N ratios in eCO_2_ rings (Brenzinger et al., 2017; Rosado-Porto et al., 2021).

Additionally, our data did not show any increase in the transcript abundance of N fixation enzymes under eCO_2_ conditions, in contrast to the reports from He et al. (2010, 2014); Xu et al. (2013); Xiong et al. (2015), and Yu et al. (2018a,b). These results support the idea that in the Gi-FACE, the enhanced N requirements are being met through the uptake of organic sources. According to our results, these sources of organic N might have been the aromatic, sulfur, and branched-chain amino acids, since their metabolism and uptake were augmented at eCO_2_ concentrations (Figures 6A, 7).

### S metabolism at eCO_2_ concentration

Most studies about the effects of eCO_2_ on the cycling of nutrients have focused on C and N cycles; nonetheless, the effects of eCO_2_ conditions have also been assessed for other elements, including S (He et al., 2010, 2014; Yu et al., 2018; Padhy et al., 2020). There are no reports about the changes in the S cycling and metabolism in the Gi-FACE. The results in the present study exhibited alterations in the metabolism of ${\text{SO}}_{4}^{2-}$. There was a reduction in transcripts involved in the dissimilatory metabolism of ${\text{SO}}_{4}^{2-}$ reduction, evidenced by the decrease in the expression of the enzymes sulfate adenylyltransferase (Sat) and adenylylsulfate reductase (AprAB) under eCO_2_ conditions (Figures 5, 7). Similarly, the first step of the assimilatory ${\text{SO}}_{4}^{2-}$ reduction metabolism changed due to eCO_2_, comprised by the depletion in the reduction step from ${\text{SO}}_{4}^{2-}$ to APS. However, the other steps of the assimilatory ${\text{SO}}_{4}^{2-}$ reduction, from the reduction of APS up to the production of S^2−^, were increased under eCO_2_ conditions (Figure 5). This phenomenon could indicate that similar to N metabolism, due to the augmented C supply, S has also become a limiting element for the development of soil organisms. Thus, the assimilatory metabolism of S was enhanced at eCO_2_ concentrations as a response to this environmental pressure. Although there are not many reports about the effect of eCO_2_ on the S cycle, Yu et al. (2018b) have described that under eCO_2_, an increase in S cycling occurred. Likewise, Padhy et al. (2020) reported an increase in the genes of the assimilatory metabolism of S under eCO_2_ conditions.

Moreover, our data suggest that the obtention of S in the Gi-FACE is not from inorganic sources but from organic ones. This process is likely a consequence of the priming effect and the mining of S from the SOM. According to our data, one of the sources for the supply of S might be sulfur amino acids, and molecules with thiol groups, due to the metabolism of these compounds, were augmented under eCO_2_ conditions (Figures 5B, 7). Moreover, although our data did not show an overall increase in the transcripts for the SOX system for the acquisition of sulfur, a slight increase in transcript abundance of the enzyme sulfane dehydrogenase (SoxC) occurred. These data indicate that soil organisms have used organic molecules to supply the S requirements in the Gi-FACE under eCO_2_ concentrations. He et al. (2014) have reported similar results, describing an increase in the expression of *sox* genes under eCO2 conditions.

## Conclusion

Our research showed for the first time how eCO_2_ simultaneously affects the gene expression in C, N, and S cycles, and potentially affects these processes at the ecosystem scale. The lower abundance of nitrogen fixation transcripts suggests that soil microorganisms degrade the SOM to fulfill their N requirements due to a higher C supply. Likewise, an increase in transcripts for carbohydrates, amino acids, chitin, lignin, and cellulose assimilation and degradation was observed. In addition, the changes in the transcript abundance of the DNRA and denitrification shed some light on the underlying mechanisms that lead to the increase of N_2_O emissions previously reported in the Gi-FACE. Additionally, this research presents evidence for the first time that the gene expression in the S cycle from the Gi-FACE changes at eCO_2_, comprised mainly by an increase in the transcript abundance of the assimilatory ${\text{SO}}_{4}^{2-}$ metabolism. Regarding soil microbiome structure, our findings confirmed previous data on the changes of bacterial and fungal communities at eCO_2_ concentrations. In addition, the results revealed new evidence of eCO_2_ effects on the virome with still unknown effects on ecosystem processes. In summary, our findings enhance our understanding of the observed changes in greenhouse gas fluxes under eCO_2_ that result in positive feedback by increased N_2_O, CH_4_, and CO_2_ emissions and reduced CH_4_ uptake.

## Data availability statement

The datasets presented in this study can be found in online repositories. The names of the repository/repositories and accession number(s) can be found below: https://www.ncbi.nlm.nih.gov/genbank/,PRJNA810964.

## Author contributions

DR-P was responsible for the experiments, data curation, data analysis, and writing of the manuscript. SR, CM, and SS contributed to methodology, review, and editing. GM contributed to data curation, review, and editing. MD was responsible for the experiments, data curation, review, and editing. All authors contributed to the article and approved the submitted version.

## Funding

The work was supported partly by the LOEWE excellence cluster FACE2FACE of the Hessian State Ministry of Higher Education, Research and the Arts.

## Conflict of interest

The authors declare that the research was conducted in the absence of any commercial or financial relationships that could be construed as a potential conflict of interest.

## Publisher's note

All claims expressed in this article are solely those of the authors and do not necessarily represent those of their affiliated organizations, or those of the publisher, the editors and the reviewers. Any product that may be evaluated in this article, or claim that may be made by its manufacturer, is not guaranteed or endorsed by the publisher.
