# Supplementary material for: Soil metatranscriptome demonstrates a shift in C, N, and S metabolisms of a grassland ecosystem in response to elevated atmospheric CO2
Source: Front Microbiol. 2022 Aug 23;13:937021. doi: 10.3389/fmicb.2022.937021 (PMC9445814; doi:10.3389/fmicb.2022.937021)
Supplement: Supplementary file 3 [file Table_3.DOCX]

**Soil metatranscriptome demonstrates a shift in C, N and S metabolisms of a grassland ecosystem in response to elevated atmospheric CO_2_**

David Rosado-Porto, Stefan Ratering, Gerald Moser, Marianna Deppe, Christoph Müller, Sylvia Schnell

**Supplement 3**

**Tab. S3.1.** Statistics of open reading frames (ORF) per sample

| **Parameter** | **Assembly** | **Sample** | | | | | | | | | | | |
| --- | --- | --- | --- | --- | --- | --- | --- | --- | --- | --- | --- | --- | --- |
|  |  | **A1b** | **A1r** | **A2b** | **A2r** | **A3b** | **A3r** | **E1b** | **E1r** | **E2b** | **E2r** | **E3b** | **E3r** |
| Number of ORFs | 4063836 | 1252429 | 1348747 | 1354232 | 1327098 | 1203036 | 1189669 | 1298076 | 1140603 | 1212203 | 1151102 | 1023406 | 1346170 |
| Number of rRNAs | 1199550 | 561169 | 596507 | 605206 | 610204 | 539292 | 550476 | 575461 | 525604 | 523504 | 535380 | 481523 | 596213 |
| Number of tRNAs/tmRNAs | 2406 | 290 | 352 | 410 | 320 | 346 | 310 | 347 | 266 | 530 | 335 | 220 | 349 |
| ORFs by Aragorn | 2406 | 290 | 352 | 410 | 320 | 346 | 310 | 347 | 266 | 530 | 335 | 220 | 349 |
| ORFs by Prodigal | 2118684 | 435792 | 484749 | 485729 | 457541 | 434426 | 413669 | 465108 | 390792 | 475930 | 399023 | 350972 | 481077 |
| ORFs by barrnap | 1199550 | 561169 | 596507 | 605206 | 610204 | 539292 | 550476 | 575461 | 525604 | 523504 | 535380 | 481523 | 596213 |
| ORFs by blastx | 743196 | 255178 | 267139 | 262887 | 259033 | 228972 | 225214 | 257160 | 223941 | 212239 | 216364 | 190691 | 268531 |
| Orphans (no hits) | 2863032 | 1049997 | 1111118 | 1111170 | 1110950 | 983486 | 991962 | 1072083 | 957476 | 950151 | 962000 | 858729 | 1114780 |
| No tax assigned (with hits) | 92698 | 22903 | 24984 | 25212 | 24026 | 20338 | 19649 | 23618 | 19826 | 19679 | 18959 | 16957 | 25887 |
| KEGG annotations | 483556 | 59151 | 74315 | 80281 | 65614 | 79358 | 65498 | 73485 | 56315 | 106774 | 60268 | 54657 | 67321 |
| COG annotations | 1163975 | 244485 | 274630 | 279795 | 257809 | 250342 | 230279 | 264068 | 218586 | 275756 | 226172 | 189731 | 272184 |
| Pfam annotations | 302811 | 40494 | 49185 | 54870 | 44563 | 54898 | 45533 | 50010 | 38021 | 72133 | 41577 | 37508 | 46468 |
| Archea annotations | 333084 | 41456 | 51995 | 57266 | 46476 | 56981 | 46802 | 51811 | 39516 | 76761 | 42877 | 38955 | 47947 |
| Bacteria annotations | 62882 | 9310 | 10407 | 12079 | 10189 | 12398 | 10706 | 11474 | 8706 | 14505 | 9267 | 8378 | 10369 |
| Fungi annotations | 662143 | 77794 | 100628 | 105066 | 85141 | 104916 | 86794 | 95638 | 73444 | 148155 | 80230 | 73061 | 90784 |
| Protozoa annotations | 261639 | 41451 | 47870 | 52742 | 46134 | 50979 | 44565 | 49196 | 38902 | 58996 | 41823 | 34943 | 49580 |
| Virus annotations | 301867 | 46491 | 53445 | 59932 | 51683 | 57145 | 48669 | 55717 | 43323 | 66617 | 45875 | 38654 | 53007 |
